# How to Screen for Monoclonal Gammopathy in Patients With a Suspected Amyloidosis

**DOI:** 10.1016/j.jaccao.2021.07.001

**Published:** 2021-10-19

**Authors:** Samuel M. Rubinstein, Keith Stockerl-Goldstein

**Affiliations:** a University of North Carolina Lineberger Comprehensive Cancer Center, Chapel Hill, North Carolina, USA; b Division of Oncology, Washington University School of Medicine, St. Louis, Missouri, USA

**Keywords:** amyloidosis, diagnosis, screening, AL, light chain amyloidosis, ATTR, transthyretin amyloidosis, FLC, free light chain, IFE, immunofixation electrophoresis, KLR, kappa:lambda ratio, PyP, technetium pyrophosphate, SFLC, serum free light chain, SPEP, serum protein electrophoresis, UPEP, urine protein electrophoresis

Amyloidosis is a category of rare diseases that result from deposition of abnormally folded proteins into organs. Light chain amyloidosis (AL) and transthyretin amyloidosis (ATTR) are the most common forms in the United States. The diagnosis of AL amyloidosis involves establishing the presence of monoclonal protein. Cardiologists evaluating patients with concern for cardiac amyloidosis should be familiar with the concepts behind testing for monoclonal proteins. We use a case to illustrate our approach to screening for monoclonal gammopathies in patients with suspected amyloidosis and how identification of a monoclonal gammopathy changes our diagnostic approach for such patients.

## Case Description

A 77-year-old man with heart failure with preserved ejection fraction, stage 3A chronic kidney disease, type 2 diabetes mellitus, and aortic stenosis was referred to the cardiology clinic with 6 months of progressive exertional dyspnea and increased diuretic requirement. Complete blood count and differential were normal. His comprehensive metabolic panel was normal except for a stable serum creatinine measurement of 1.72 mg/dL. Troponin T and N-terminal pro–B-type natriuretic peptide levels were elevated at 0.27 ng/mL and 5,114 pg/mL, respectively. An echocardiogram was performed before referral. Left ventricular ejection fraction was 58%, with marked concentric left ventricular hypertrophy (septal wall thickness: 1.9 cm), and left ventricular global longitudinal strain of –14.7% with apical sparing strain pattern. These findings were concerning for an amyloidosis.

## Introduction

Amyloidoses are diseases in which misfolded proteins deposit in organs, interfering with normal function. Cardiac involvement is common and can result in arrhythmias or clinical heart failure events, which are often fatal (1). The 2 most common types of cardiac amyloidosis in the United States are AL amyloidosis and ATTR amyloidosis. The treatment paradigm for both disorders is to reduce bloodstream concentrations of amyloidogenic protein, and the specific therapeutic approach depends on the subtype of amyloidosis. AL amyloidosis is typically treated with chemotherapy targeted at clonal populations of plasma cells that are responsible for producing amyloidogenic light chains. By contrast, ATTR amyloid fibrils are misfolded monomers of transthyretin, a tetrameric protein produced by the liver that binds thyroid hormone. The most widely used ATTR amyloidosis therapeutics either reduce transthyretin production (inotersen and patisiran) or stabilize the tetramer (tafamidis) (2). Given the divergent therapeutic approaches, determining the subtype of amyloidosis is of paramount importance. In patients with suspected amyloidosis, both AL and ATTR amyloidosis should be considered.

### Monoclonal protein identification

Diagnosing AL amyloidosis requires showing the presence of a clonal plasma cell dyscrasia, which starts with monoclonal protein evaluation (3). The rationale for recommended monoclonal protein evaluation is highlighted in Table 1. This monoclonal protein can be intact immunoglobulin or immunoglobulin fragments, such as free light chains (FLC) or less-commonly free heavy chains. The most widely used laboratory test to identify monoclonal protein is serum protein electrophoresis (SPEP). Serum is loaded onto a medium to which an electrical current is applied, separating constituent proteins into 5 distinct regions based on electrical charge and size: albumin, α1, α2, β, and γ. As most antibodies migrate into the γ region, the monoclonal proteins are generally identifiable as a sharp peak in the γ region of the SPEP, referred to as an “M spike.” SPEP neither establishes the subtype of monoclonal protein nor confirms that an M spike represents monoclonal protein. This requires immunofixation electrophoresis (IFE), which involves exposing serum to antibodies against various heavy and light chain subtypes. IFE is often performed reflexively in patients with abnormal SPEP, but if not, both SPEP and IFE need to be obtained.

**Table 1:** Monoclonal Protein Assessment Components

	Purpose	Limitations
SPEP/IFE	Shows monoclonal intact antibodies or light chains	Both SPEP and IFE need to be performed; not all laboratories reflexively perform both tests Negative in 30% of patients with light chain amyloidosis
SFLC	Shows excess free light chain production	Excess light chain production may be polyclonal or monoclonal Interpret minor abnormalities with caution in patients with renal disease, other inflammatory conditions
Urine protein electrophoresis/IFE	Establishing clonality in patients with subtle SFLC abnormalities	Rarely positive in isolation Collection is cumbersome

IFE = immunofixation electrophoresis; SPEP = serum protein electrophoresis; SFLC = serum free light chain.

**Figure 1:**
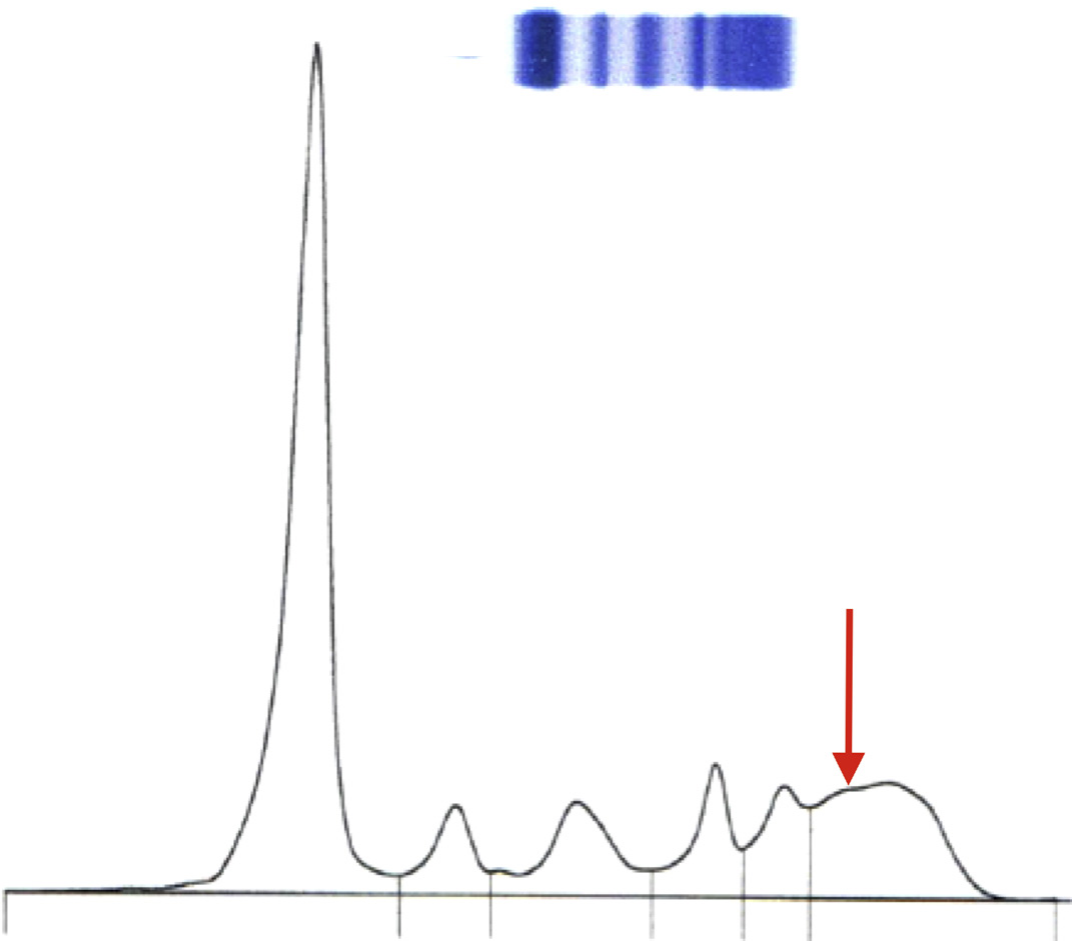
SPEP/IFE in a Patient With AL Amyloidosis No quantifiable M spike is seen, but a subtle abnormality in the gamma region was identified **(arrow)**. Trace monoclonal free lambda light chain was identified on immunofixation. AL = light chain amyloidosis; SPEP/IFE = serum protein electrophoresis and immunofixation.

Although SPEP/IFE yields an abnormal result in most patients with monoclonal proteins, it has limitations. The burden of clonal plasma cells in patients with AL amyloidosis is typically low, meaning that the amount of monoclonal protein produced can be difficult to measure. The clonal plasma cells in patients with AL amyloidosis often produce monoclonal FLC only; because FLC are smaller than intact immunoglobulins, they can migrate to SPEP regions other than γ or can be excreted in the urine. Due to these factors, an M spike cannot be identified on SPEP in up to 30% of patients with AL amyloidosis (Figure 1) (4). Additional testing beyond SPEP/IFE is therefore required to exclude a monoclonal protein in patients with suspected amyloidosis.

Serum free light chain (SFLC) measurements must also be obtained. Typically, concentrations of both light chain subtypes (kappa and lambda) and the kappa:lambda ratio (KLR) are reported. Adding SFLC to SPEP/IFE can significantly increase the ability to identify serum monoclonal protein in patients with AL amyloidosis, with sensitivity up to 99% for both assays in combination (4). Abnormal SFLC results do not always indicate the presence of monoclonal protein. Symmetric elevations in kappa and lambda light chain (with normal KLR) do not establish monoclonal protein and can be characteristic of nonspecific inflammatory states. Because light chains are renally excreted, patients with chronic kidney disease have impaired light chain clearance, with worsening in the normally more efficient kappa (compared with lambda) light chain clearance. Because kappa light chains are also typically produced at a higher rate than lambda, modestly elevated KLRs in patients with chronic kidney disease may be due to perturbed balance of clearance and production.

In addition to SPEP/IFE and SFLC, 24-hour urine protein electrophoresis (UPEP) and IFE should be obtained. Although 24-hour UPEP/IFE has low sensitivity alone, it is useful to show the presence of clonal light chains when the only evidence of monoclonal protein is a subtle SFLC abnormality. In such patients, 24-hour UPEP/IFE may be the only test that definitively identifies a monoclonal protein. Finally, although a detailed discussion is beyond the scope of this primer, 24-hour UPEP/IFE is useful in determining the etiology of renal injury in patients presenting with new renal insufficiency and a monoclonal gammopathy. As such, even if a monoclonal protein has already been identified, 24-hour UPEP/IFE may have additional management implications and should be obtained.

### Key points

• SPEP/IFE alone are insufficiently sensitive to screen for monoclonal protein in all patients with suspected amyloidosis.
• Serum FLC and 24-hour UPEP/IFE should be obtained in addition to SPEP/IFE in patients with suspected AL amyloidosis.

## Case Description Continued

Additional laboratory studies were obtained. SPEP was performed and revealed an M spike of 0.1 g/dL, with IFE identifying monoclonal immunoglobulin G kappa. Serum FLC revealed an elevated kappa light chain of 145.7 mg/L (normal: 3.3-19.4 mg/L) and normal free lambda of 8.35 mg/L (normal: 5.7-26.3 mg/L), with an abnormal KLR of 17.4 (normal: 0.26-1.65). Twenty-four-hour UPEP revealed 576 mg/d of protein, predominantly albumin with negative IFE. Fat pad aspiration was negative for amyloid deposition. This finding prompted hematology referral. Results of a bone marrow biopsy revealed a normocellular marrow with trilineage hematopoiesis and no increase in plasma cells. A technetium pyrophosphate (PyP) scan was performed, revealing significant tracer uptake throughout the myocardium with activity much greater than bone. Genotyping was negative for transthyretin gene mutations.

## How Identifying a Monoclonal Protein Changes the Diagnostic Approach

Although identification of a monoclonal plasma cell disorder is necessary to establish the diagnosis of AL amyloidosis, it is not sufficient. As this case illustrates, patients with abnormal FLC may not always have clonal populations of plasma cells on bone marrow biopsy, due to sampling error in the setting of a low overall burden of clonal plasma cells or extramedullary disease.

It is important to keep in mind that ATTR amyloidosis and monoclonal gammopathies are often coincident, given shared associations with age and Black race. As such, many patients with ATTR amyloidosis have unrelated monoclonal gammopathies, although the literature may overstate the frequency of this coincidence due to the inclusion of minor SFLC abnormalities as monoclonal gammopathies (5). Identification of a monoclonal protein therefore needs to be followed with additional evaluation to determine that a patient has AL amyloidosis.

The results of PyP scans should be interpreted with caution in the context of a monoclonal protein. This is especially true in the context of a positive PyP scan, as these scans cannot be used to distinguish between AL amyloidosis and ATTR amyloidosis. Although the sensitivity of PyP for a diagnosis of ATTR amyloidosis in patients without monoclonal protein is nearly 100%, as many as 10% of patients with positive PyP scans and monoclonal proteins may be found to have AL amyloidosis on subsequent endomyocardial biopsy (6). Because the current patient has an elevated kappa light chain, the positive PyP result does not establish the subtype of amyloidosis, and a PyP scan should not have been performed in this patient due to the presence of monoclonal protein.

Given the propensity of patients with amyloidosis to develop periprocedural complications, it is reasonable to pursue the least invasive diagnostic evaluation possible. Abdominal fat pad aspiration is commonly performed in this context and has been shown to have sensitivity in excess of 80%, particularly when combined with bone marrow aspiration (7). However, even in prospective studies, significant proportions of patients with confirmed AL amyloidosis have negative fat pad aspirations, and sensitivity is lower in centers that do not perform fat pad aspirations in high volumes, and much lower in patients with ATTR amyloidosis (8). Although positive fat pad aspirations are useful, negative results should not be construed as definitive. If this approach fails to identify the amyloidosis subtype, involved organ biopsy is the gold standard and may be necessary.

### Key points

• Presence of monoclonal protein does not establish a diagnosis of AL amyloidosis.
• PyP scans should not be obtained in patients with monoclonal protein.
• Negative fat pad aspiration does not exclude systemic amyloidosis.

## Conclusions

The patient underwent an endomyocardial biopsy. Fibrillary typing by mass spectrometry was performed, identifying the subtype of amyloidosis as ATTR amyloidosis. The patient was started on tafamidis.

In summary, all patients with suspected amyloidosis should be evaluated for the presence of a clonal plasma cell population, which starts with thorough assessment for monoclonal protein: SPEP/IFE, serum FLC, and 24-hour UPEP/IFE. Although the presence of monoclonal protein can suggest AL amyloidosis, establishing this diagnosis requires both proving the presence of monoclonal protein and showing that the amyloidosis is the AL subtype. Although noninvasive evaluation is helpful, histopathological confirmation of the diagnosis is often necessary.

## Funding Support and Author Disclosures

Dr Rubinstein has received advisory fees from Roche Diagnostics and Sanofi. Dr Stockerl-Goldstein has reported that he has no relationships relevant to the contents of this paper to disclose.
